# Rating of Everyday Arm-Use in the Community and Home (REACH) Scale for Capturing Affected Arm-Use after Stroke: Development, Reliability, and Validity

**DOI:** 10.1371/journal.pone.0083405

**Published:** 2013-12-16

**Authors:** Lisa A. Simpson, Janice J. Eng, Catherine L. Backman, William C. Miller

**Affiliations:** 1 Graduate Program in Rehabilitation Sciences, University of British Columbia, Vancouver, Canada; 2 Rehabilitation Research Program, GF Strong Rehab Center, Vancouver Coastal Health, Vancouver, Canada; 3 Department of Physical Therapy, University of British Columbia, Vancouver, Canada; 4 Department of Occupational Science and Occupational Therapy, University of British Columbia, Vancouver, Canada; Innsbruck Medical University, Austria

## Abstract

**Objective:**

To develop a brief, valid and reliable tool [the Rating of Everyday Arm-use in the Community and Home (REACH) scale] to classify affected upper limb use after stroke outside the clinical setting.

**Methods:**

Focus groups with clinicians, patients and caregivers (n = 33) and a literature review were employed to develop the REACH scale. A sample of community-dwelling individuals with stroke was used to assess the validity (n = 96) and inter-rater reliability (n = 73) of the new scale.

**Results:**

The REACH consists of separate scales for dominant and non-dominant affected upper limbs, and takes five minutes to administer. Each scale consists of six categories that capture ‘no use’ to ‘full use’. The intraclass correlation coefficient and weighted kappa for inter-rater reliability were 0.97 (95% confidence interval: 0.95–0.98) and 0.91 (0.89–0.93) respectively. REACH scores correlated with external measures of upper extremity use, function and impairment (rho = 0.64–0.94).

**Conclusions:**

The REACH scale is a reliable, quick-to-administer tool that has strong relationships to other measures of upper limb use, function and impairment. By providing a rich description of how the affected upper limb is used outside of the clinical setting, the REACH scale fills an important gap among current measures of upper limb use and is useful for understanding the long term effects of stroke rehabilitation.

## Introduction

Measurement of functional recovery following stroke is an important aspect in the assessment of stroke care. In addition, it is critical to determine whether patients incorporate the functional improvements they gained over rehabilitation into their daily lives; otherwise, any gains made may be lost. This “use it or lose it” phenomenon is particularly evident following motor rehabilitation of the upper extremity (UE) following stroke [1].

To date, there is no simple tool that describes the manner in which the affected upper limb is used in the community and home setting. In contrast, Perry et al. (1995) [2] developed a 6-category scale to classify walking ability in the home and community and this scale has received high utility by stroke researchers and clinicians. The Motor Activity Log (MAL) (14 or 30-item) [3], [4] and accelerometry [5] are two current tools that assess affected arm use. While large MAL scores indicate greater quantity and quality of affected arm use, the scores do not inform clinicians how patients are using their upper limb in daily living (e.g., uses hand only for stabilisation, and not manipulation). Accelerometry, which measures the quantity of affected arm use, does not capture the type of activity the arm or hand is performing.

A measure of real-world arm use can guide realistic treatment goals, activity prescription and evaluation of patient-oriented outcomes; thereby maximizing the potential for lasting functional gains. The aim of this study was to 1) develop a classification scale called the Rating of Everyday Arm-use in the Community and Home (REACH) scale that captures affected upper limb use outside of the clinical setting and 2) to assess the inter-rater reliability and validity of the new scale.

## Methods

### Ethics statement

The study was approved by the University of British Columbia Clinical Research Ethics Board and participants provided informed written consent.

### Development of the REACH scale

A multiphase process was used to develop a classification scale to distinguish between different levels of affected UE use that are meaningful to individuals with stroke, caregivers and clinicians.

### Content generation by focus groups

First, to derive patient and clinician driven descriptions of affected UE use, a combination of 7 focus group sessions and 3 individual interviews were conducted. Thirteen experienced clinicians who worked in the community or an outpatient setting from the following disciplines were recruited: Physical Medicine, Occupational Therapy, Physical Therapy, Nursing and Recreational Therapy (**Table 1**). Sixteen individuals with stroke with a range of impairment level (tested by active range of motion), who lived in the community and were at least 6 months post stroke participated in the focus group sessions or interviews (**Table 1**). Four caregivers who assisted a participant with stroke with daily or instrumental daily activities also took part (**Table 1**). One facilitator with one assistant (both rehabilitation scientists with clinical backgrounds) led the focus groups using separate discussion guides for each participant group. Sessions with clinicians centred on the number and content of scale categories that best captured a progression from “no use” to “full use”. Sessions with patients/caregivers gathered patient descriptions of use and meaningful change in use. Interviews were conducted with individuals unable to attend group sessions or who had expressive aphasia which limited their communication in a group setting (n = 3). All sessions were audio recorded, transcribed and analysed using qualitative descriptive analysis [6]. Themes were organized based on the types of questions presented to the groups. Findings from each participant group (clinician; patient/caregiver) were compared to identify unique and common themes.

**Table 1:** Focus group participant characteristics

Characteristics	Individuals with StrokeN = 16	CaregiversN = 4	Healthcare ProvidersN = 13
Female, N (%)	9 (56%)	2 (50%)	13 (100%)
Age, mean (SD)	65.3 (7.0)		
Years post stroke (SD)	7.8 (3.3)		
Dominant hand affected, N (%)	7 (44%)		
AHA stroke functional classification, N (%)			
I	10 (63%)		
II	2 (12%)		
III	3 (19%)		
IV	1 (6%)		
Active Range of Motion, N (%)			
None	4 (25%)		
Shoulder only	5 (31%)		
Shoulder and hand	7 (44%)		
Years practicing, mean (SD)			16.3 (9.7)
Profession (N)			
Occupational Therapist			5
Physiotherapist			4
Physiatrist			1
Nurse			2
Recreational Therapist			1

AHA: American Heart Association.

### Scale refinement

Focus group findings were supplemented with information from the literature to generate an initial draft of the REACH scale. Feedback sessions with clinicians (n = 9) and pilot testing on individuals with stroke (n = 8) were conducted to refine the scale and ensure content relevance, question and scoring clarity and minimization of respondent bias.

### Evaluation of reliability and validity

To assess the measurement properties of the REACH scale, 96 individuals who had experienced a stroke and with weakness in one UE at least six months prior to testing were recruited from community stroke recovery groups and former inpatients from a local rehabilitation hospital. Individuals were excluded if they had a neurological condition other than stroke or a musculoskeletal condition that affected use of either UE (e.g., fracture or arthritis). Individuals with expressive aphasia were included in the study if a family member was present during testing or when individuals were able to provide consistent yes/no responses as confirmed by their speech language pathologist.

Based on the content of the REACH scale, we a priori hypothesized that REACH scale scores would be strongly correlated to current measures of UE use, UE function and UE impairment. The Motor Activity Log-14 (MAL-14) Amount of Use scale^3^ and average daily activity counts of the affected UE captured by Actical™ accelerometers were used to assess UE use [7], [8]. Self-perceived UE function was assessed with the Stroke Impact Scale-hand subscale (SIS-hand) [9] and the Action Research Arm Test [10] was used to capture UE functional performance. Finally, the Chedoke-McMaster Stroke Assessment (arm and hand subscale out of 14, shoulder pain subscale out of 7) [11] were used to assess UE motor impairment and shoulder pain.

A cross-sectional design was used to examine the relationships between REACH scale scores and the comparators. Except for the accelerometers, all assessments (n = 96) were administered during a single test session by two trained clinicians (Occupational Therapist, Physical Therapist). A subsample of 78 participants willing to wear the accelerometers were shown how to wear the monitor on the stroke-affected wrist during waking hours for three consecutive days. These participants took home an illustrated pamphlet to reinforce the instructions. To account for differences in wake and sleep times, the average daily activity counts of the affected UE were calculated across the three consecutive days. Individuals were excluded from the accelerometer analysis if their data displayed irregular or unexplainable activity (e.g., 24 hours of constant activity, large random spikes of activity potentially indicative of shaking the monitor). A total of 68 individuals were retained in the final accelerometer analysis.

Seventy-three individuals agreed to a second test session to examine the inter-rater reliability of the REACH scale. Testing sessions were separated by a mean period of 7 days (range 3–21 days) to minimize administrator and respondent memory bias. Change in UE use among individuals greater than six months post stroke was not expected within time frames of 1–3 weeks [12]. Two different raters were used to assess inter-rater reliability whereby the rater used at the first test session was different than the rater used at the second test session. At the second testing, the testers were blind to the results of the first evaluation.

The spearman rank correlation coefficient (rho) was used to examine correlations between REACH scale scores and the external measures. Inter-rater reliability was assessed with the intraclass correlation coefficient (ICC) (two-way random effects model with absolute agreement) [13] and the linear weighted kappa (Κ_w_) [14]. Scatterplots were used to visually examine the nature of the relationships between the REACH scale and external measures, as well as the relationships between the two raters. In addition, the standard error of measurement (SEM) and the smallest real difference calculated at 95% confidence level (SRD) were used to estimate important change in the REACH score.

Sample size estimations for this study were based on the desired magnitude and precision of the inter-rater reliability ICC. To detect an ICC of 0.80 with a 95% confidence interval of 0.25, a sample size of 37 individuals was required [15]. At a power of 80% and an alpha of 0.05, a sample size of 37 surpasses the 28 individuals required to detect hypothesized correlations of at least 0.50 between the REACH scale scores and the external measures [16]. ****

## Results

### Development of the REACH Scale

The multiphase process which consisted of focus groups, literature review, feedback consultations and preliminary testing on individuals with stroke resulted in the development of the final REACH scale.

**Figure 1:**
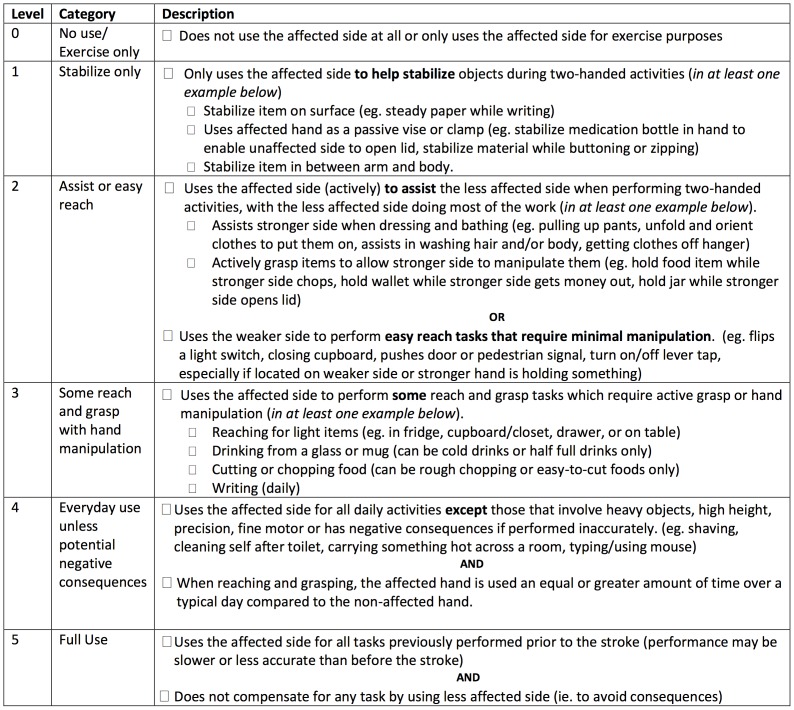
Checklist for REACH scale dominant side affected.

**Figure 2:**
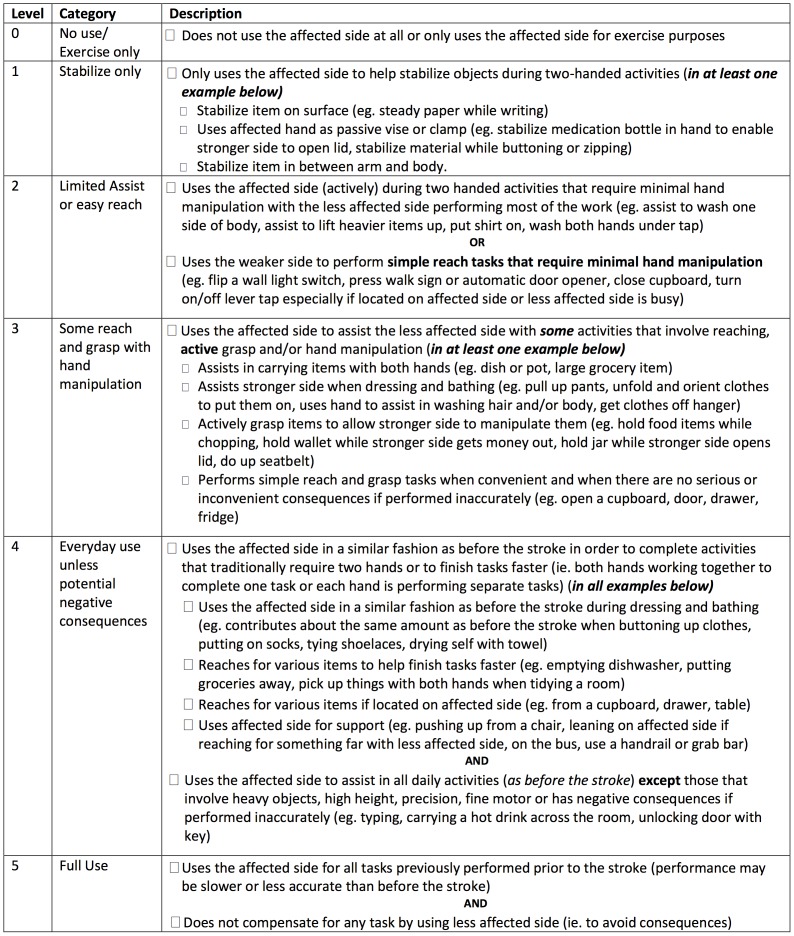
Checklist for REACH scale non-dominant-side affected.

**Figure 3:**
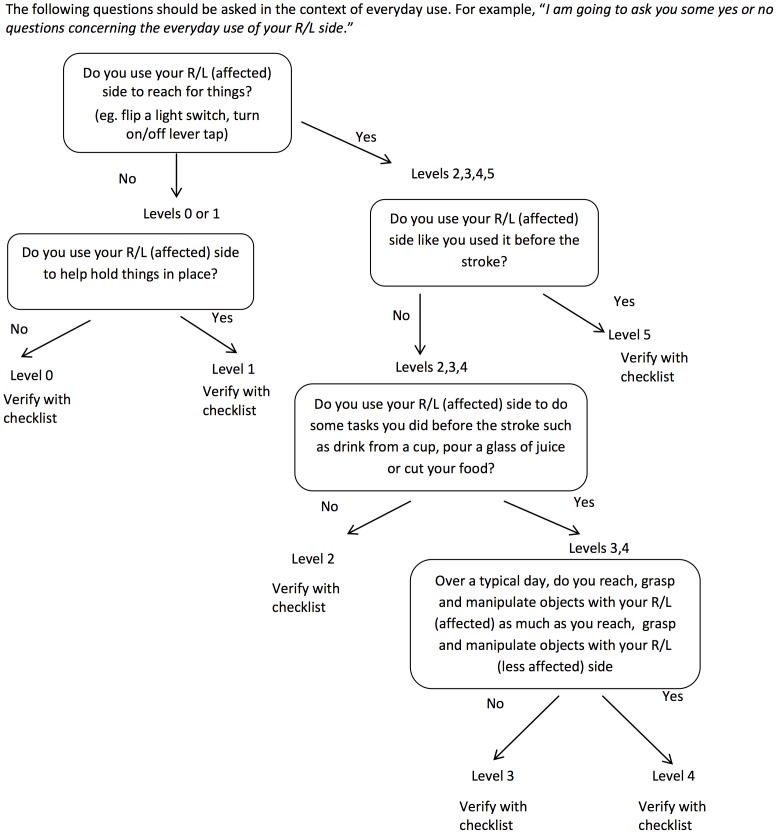
Algorithm for REACH scale dominant side affected.

**Figure 4:**
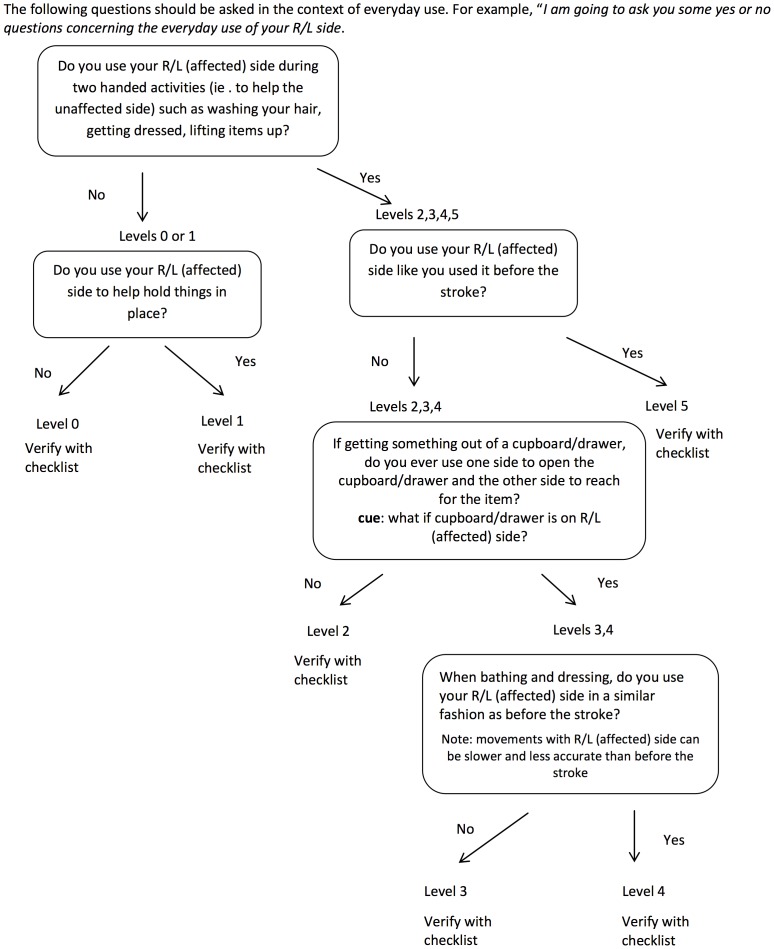
Algorithm for REACH scale non-dominant side affected.

The REACH scale consists of two separate classifications scales for people who had their dominant (Figure 1) and non-dominant side (Figure 2) affected by the stroke. Both scales classify use into six categories that capture a similar progression from “no use” to “full use”. Full use is defined relative to the patient’s affected UE use prior to the stroke. The REACH scale consists of the six classification levels and their respective attributes, and an algorithm (Figures 3,4) is provided to narrow down a patient’s appropriate level.

### Focus Group Findings and Literature Review

Comparison of the focus group findings across the patient and clinician groups revealed both common and unique themes that were incorporated into the final REACH scale. First, both patient and clinician groups identified hand dominance as an important factor that influences the role of the affected UE and the circumstances under which it is used. This finding was also supported by literature review that revealed differences in patterns of affected UE use among dominant and non-dominant side affected patients [17], [18]. Separate scales for people who had their dominant and non-dominant side affected were thus created.

The formulation of distinct REACH categories was based on the combination of the following focus group themes and assumptions of typical UE use drawn from the literature. Patient and clinicians groups identified the following common themes: 1) roles of the affected UE during everyday use (e.g., stabilizing or manipulating objects); 2) circumstances when the affected UE was used (e.g., during two-handed activities, when the unaffected UE was occupied) and 3) factors that impact UE use (e.g., motor impairment, confidence in the ability of the UE to perform a specific task). In addition, the following assumptions concerning typical arm use were adopted from Barreca et al. [19]: 1) efficient performance of daily home and community activities involves the cooperative use of both upper limbs; 2) both upper limbs fulfill the following roles during typical performance of daily activities: reach and grasp, stabilize and manipulate objects; and 3) typical use of the upper limbs involves an interaction with objects of various sizes, weights and locations.

Salient themes that were unique to either the clinician or patient groups were also incorporated into the final REACH scale. For instance, the majority of the clinicians stated that five categories would provide adequate discrimination between “no use” and “full use”. However, a wide variation in the types of activities and roles of the affected UE during use was reported among patients with more severe UE impairment. This finding supported the addition of an extra level at the lower end of the REACH scale resulting in the six categories. Finally, clinicians and patients disagreed on the manner in which amount of use should be captured. Amount of use was identified as an important dimension for inclusion in a scale that captures UE use according to the clinicians. Many clinicians recommended using percentage values to differentiate between different levels of affected UE use (e.g., greater or less than 50% of pre-stroke use). In contrast, patients demonstrated difficulty interpreting percentage values during focus group discussions and preliminary testing of the scale. Ultimately, percentage values were excluded and descriptors were selected consistent with patient preferences.

### Evaluation of reliability and validity

Demographic and clinical characteristics of the sample used to evaluate the measurement properties of the REACH scale are in **Table 2**. In summary, individuals with stroke were in the chronic phase (mean 7.8 years post stroke), presented with mild to moderate stroke severity (National Institute of Health Stroke Scale) and represented the full range of UE impairment levels from mild to severe (Chedoke-McMaster arm and hand subscale range 2–14). Just over half of the sample was dominant side-affected.

**Table 2:** Participant characteristics (N = 96 except for activity counts where N = 68).

Characteristic	
**Age**, mean (SD; range)	64.4 (11.7; 32–96)
**Females**, N (%)	47 (49.0%)
**Years post stroke**, mean (SD; range)	7.0 (5.4; 0.5–23.3)
**Right side affected**, N (%)	52 (54.2%)
**Dominant Side Affected**, N (%)	52 (54.2%)
**Living Situation**, N (%)	
Spouse/Family	62 (64.6%)
Alone	29 (30.2%)
Assisted Living	5 (5.2%)
**Years of Education**, mean (SD; range)	14.7 (2.9;4–21)
**Previous Inpatient Rehabilitation**, N (%)	93 (96.9%)
**NIHSS**, mean (SD; range)	3.9 (3.0;0–12)
**MoCA^a^**, mean (SD; range)	26.0 (3.5;15–30)
**UE use measures**	
MAL mean (SD; range)	1.9 (1.8; 0–5)
Affected UE Activity Counts mean (SD; range)	108,540 (80,325; 4,353–324,593)
**UE function measures**	
ARAT, mean (SD; range)	29.1 (24.0; 0–57)
SIS-hand, mean (SD; range)	38.1 (33.8; 0–100)
**UE impairment measures**	
Chedoke-arm and hand, mean (SD; range)	9.0 (4.5; 2–14)
Chedoke-shoulder pain, mean (SD; range)	5.8 (1.5; 1–7)

–42); MoCA: Montreal Cognitive Assessment (0–30); ^a^17 people were not included in the calculation of the mean MoCA score due to aphasia; UE: upper extremity; MAL: Motor Activity Log (0–5); UE: upper extremity; ARAT: Action Research Arm Test (0–57); SIS-hand: Stroke Impact Scale-hand scale (0–100); Chedoke-arm and hand: Chedoke-McMaster arm and hand subscale (2–14); Chedoke-shoulder pain: Chedoke-McMaster shoulder pain subscale (1–7). NIHSS: National Institute of Health Stroke Scale (0

The mean REACH sample score was very similar across the non-dominant (2.6) and dominant affected groups (2.3). All categories, from “no use” to “full use” were represented in the sample; however the greatest number of individuals used their affected UEs to perform some reach and grasp tasks that require hand manipulation (Level 3) (**Table 2**). The average time to administer the REACH scale in this sample was five minutes.

**Figure 5:**
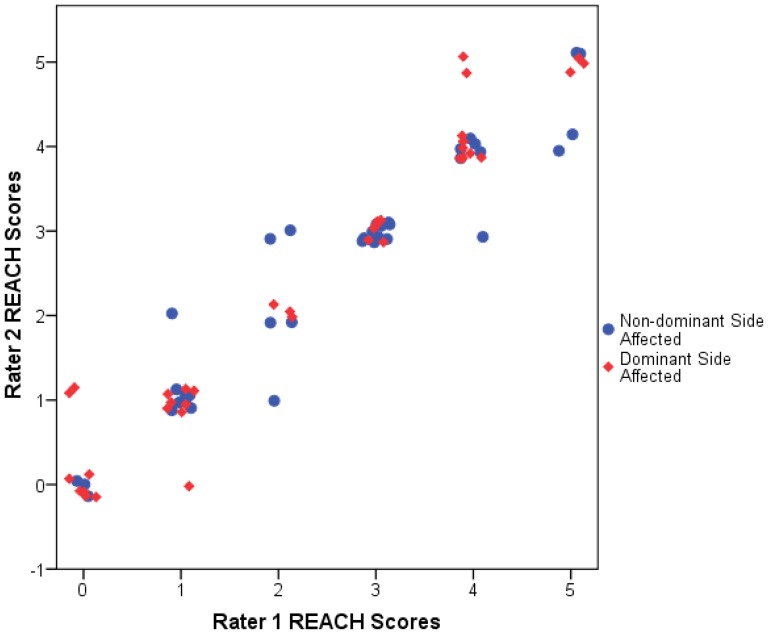
Plot of rater agreement. The plot shows the REACH scores of Rater 1 versus Rater 2 for each participant. The plot includes a horizontal jitter to ensure visibility of data points at identical locations.

The inter-rater reliability scatterplot demonstrated that the dominant and non-dominant affected subjects had similar relationships between Rater 1 and Rater 2 with similar slopes and range of data (**Figure 5**). Thus, all participants (dominant and non-dominant affected subjects) ratings were combined to assess reliability. The ICC (2,1) for the REACH scale was 0.97 (95%CI: 0.95–0.98, p<0.001) and the Κ_w_ was 0.91 (95%CI: 0.89–0.93, p<0.001). The SEM was 0.27 and the SRD was 0.75. These values represent 4.5% and 12.5% of the 6-category REACH score respectively. Post-hoc analysis justified the collapsing of the data as the absolute differences of the reliability coefficients (ICC or weighted kappa) between the dominant and non-dominant affected scales were less than 0.05.

**Figure 6:**
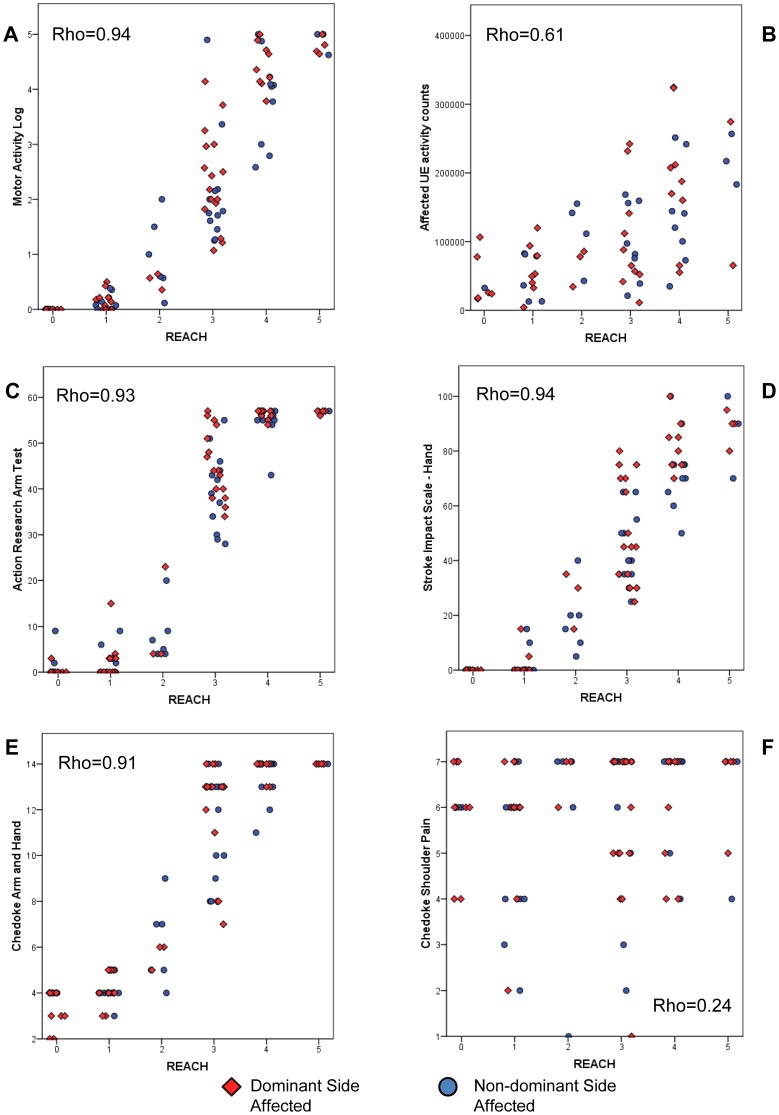
Scatterplots of REACH scale scores with external measures of upper extremity (UE) use, function and impairment. Plots A, B show relationship between REACH scores and measures of UE use. Plots C, D show relationship between REACH scores and measures of UE function. Plots E, F show relationship between REACH scores and measures of UE impairment. The plots include a horizontal jitter to ensure visibility of data points at identical locations.

Similarly, scatterplots between the REACH scale and external measures demonstrated that the dominant and non-dominant affected subjects had similar relationships (same slopes and range of data, **Figure 6A-F**), and thus, the data were combined. **Table 3** displays correlation coefficients and **Figure 6** displays the scatterplots of the relationships. Except for the relationship between the REACH scale and shoulder pain, the REACH scale was correlated to other measures of UE use, UE function and UE impairment. The relationships were particularly strong between the REACH scale and the MAL, SIS, ARAT and Chedoke-McMaster arm and hand subscale (rho = 0.87–0.94, p<0.001) (**Table 3**). The correlation between the REACH scale and the Chedoke-shoulder pain subscale was low (rho = 0.24) but statistically significant (p = 0.02). Inspection of the scatterplots revealed interesting differences between the distribution of the REACH scale scores in relation to the MAL, ARAT and the Chedoke-arm and hand subscale (**Figure 6** **A, C, E**). These graphs show greater frequency of REACH scores at the lower ends and higher ends of use, resulting in a slight curvilinear “S” relationship between the REACH scores and these impairment and functional scales.

**Table 3:** Correlations between REACH scores and established external measures.

Outcome Measure	Spearman rank correlation coefficient
**UE use measures**	
MAL (n = 96)	rho = 0.94, p<0.001
Affected UE Activity Counts (n = 68)	rho = 0.61, p<0.001
**UE function measures**	
ARAT (n = 96)	rho = 0.93, p<0.001
SIS-hand (n = 96)	rho = 0.94, p<0.001
**UE impairment measures**	
Chedoke-arm and hand (n = 96)	rho = 0.91, p<0.001
Chedoke-shoulder pain (n = 96)	rho = 0.24, p = 0.02

UE: upper extremity; MAL: Motor Activity Log; UE: upper extremity; ARAT: Action Research Arm Test; SIS-hand: Stroke Impact Scale-hand scale; Chedoke-arm and hand: Chedoke-McMaster arm and hand scales; Chedoke-shoulder pain: Chedoke-McMaster should pain scale.

Finally, post-hoc analysis justified the collapsing REACH data from dominant and non-dominant hand scales because the absolute differences in the correlation coefficients were less than 0.03, except for shoulder pain, where the difference was 0.18.

## Discussion

The REACH scale was created from a multiphase process that considered UE use from the perspective of patients and clinicians. The resulting scales capture the progression from no use to full use for both the dominant and non-dominant UE. Studies have observed different patterns of use depending on whether the dominant or non-dominant side was affected [17], [18]. The REACH scores were not evenly distributed across our sample. The number of dominant and non-dominant individuals classified into Level 3 was particularly high. The observed category frequencies represent the underlying distribution of affected UE use in this chronic population (mean of 7 years post stroke), while we might expect more individuals to move through other levels during the acute phase of stroke recovery. Future studies using the REACH scale to classify UE use over time would shed light on the frequency of different categories as individuals move from more acute to more chronic phases of recovery.

Evaluation of inter-rater reliability indicated a highly reliable scale with a weighted kappa of 0.91 (lower bound of 0.89) and ICC value of 0.97 (lower bound of 0.95). It should be noted that these reliability values represent conservative estimates as they capture both the effect of different raters and time. This design was selected in order to minimize rater and respondent memory bias and to reflect a realistic clinical scenario where different clinicians often perform baseline and follow up assessments.

Moderate to strong relationships were observed between the REACH scale scores and external measures of UE impairment, function and use (rho = 0.61–0.94). One exception was the weak relationship between the REACH scale and the Chedoke-shoulder pain subscale. One explanation for these findings is the large number of people in our study with no or little shoulder pain.

The REACH scale scores were more strongly correlated to the MAL than the affected UE activity counts. The stronger relationship with the MAL likely occurs because the MAL captures functional activity alone, while activity counts capture both functional and non-functional activity. In addition, both the REACH and MAL are self-report and may be subject to similar biases. The correlations between the REACH scale scores and measures of UE function and motor impairment (rho = 0.91–0.94) were higher than those found in the literature. Cited correlation coefficients between UE use (captured by the MAL and accelerometry) and UE function or UE impairment range from 0.40–0.82 [4], [12], [20]–[25] and 0.54–0.85 [8], [23], [26] respectively. The high correlations in the present study may arise from the nature of the REACH scale, which progresses from simple to complex tasks requiring greater motor function, whereas the MAL and accelerometry measures the frequency of movement.

Taking only five minutes to administer, the REACH scale is a brief tool that provides a rich description of affected UE activity outside of the clinical setting; this is paramount for understanding long term effects of rehabilitation following stroke. Exploration of patients’ perceptions of meaningful change in the current study contributed to development of the six categories of the REACH scale. Of importance, we obtained an SRD value of 0.75, which suggests that a change in the REACH scale of one category represents a meaningful change in affected UE use. While the REACH scale fills an important gap among current measures of UE use, further studies that examine the change in REACH scores over time or as a result of different treatments are needed.

## Summary/Conclusions

This study provides strong evidence to support the reliability and validity of the REACH scale; a quick-to-administer classification tool that captures UE use outside of the clinical setting.
